# Unusual denture-associated oral squamous papilloma with koilocytosis: a case report and literature review

**DOI:** 10.3389/fdmed.2026.1871334

**Published:** 2026-06-15

**Authors:** Azza Sioufi, Maya Al-Jokhadar, Faisal Mehsen Alali, Bassel Tarakji, Mohammad Zakaria Nassani, Shahed Kuraitby, Mai Adnan Gaizeh Al-Hallak, Rafif Alshenaiber, Hamod Alqahtani, Hunaida Khaled Tayeb, Asim Abdullah Aldhali, Anas Alsalhani

**Affiliations:** 1 Aljazeera Hospital, Riyadh, Saudi Arabia; 2 Department of Oral Histology and Pathology, College of Dentistry, Arab International University, Daraa, Daraa Governorate, Syria; 3 Department of Oral Maxillofacial Surgery and Diagnostic Sciences, College of Dentistry, Prince Sattam Bin Abdulaziz University, Al-Kharj, Saudi Arabia; 4 Department of Oral Pathology, Faculty of Dentistry, University of Aleppo, Aleppo, Syria; 5 Department of Restorative and Prosthetic Dental Sciences, College of Dentistry, Dar Al Uloom University, Riyadh, Saudi Arabia; 6 Department of Removable Prosthodontics, Faculty of Dentistry, University of Aleppo, Aleppo, Syria; 7 Department of Oral Medicine, Faculty of Dental Medicine, Damascus University, Damascus, Syria; 8 Department of Oral Medicine, College of Dentistry, Arab Internationa University, Daraa, Daraa Governorate, Syria; 9 Prosthetic Dental Sciences Department, Prince Sattam bin Abdulaziz University, Al-Kharj, Saudi Arabia; 10 Department of Prosthetic Dental Sciences, College of Dentistry, Prince Sattam Bin Abdulaziz University, Al-Kharj, Saudi Arabia; 11 Oral and Maxillofacial Prosthodontics Department, Faculty of Dentistry, King Abdulaziz University, Jeddah, Saudi Arabia; 12 Department of Dentistry, Vision Colleges, Riyadh, Saudi Arabia; 13 Department of Histology and Pathology, Faculty of Dentistry, University of Hama, Hama, Syria

**Keywords:** alveolar mucosa, CARE guidelines, case report, denture stomatitis, human papillomavirus, koilocytes, literature review, oral squamous papilloma

## Abstract

**Background:**

Oral squamous papilloma (OSP) is a benign epithelial proliferation commonly linked to human papillomavirus (HPV) and usually affects the palate, tongue and lips. Anterior mandibular alveolar mucosa involvement is uncommon, and overlap between koilocytic HPV-suggestive change and chronic denture-related trauma is rarely documented. A review of the literature indicates limited documentation of such overlapping clinicopathological features at this anatomical site.

**Case presentation:**

A 49-year-old non-smoking male with absence of alcohol consumption history, presented with a 12-month painless, slowly enlarging papillary mass on the anterior mandibular alveolar mucosa beneath a poorly maintained removable denture. Intra-oral examination showed firm papillary folds of near-normal color and an adjacent traumatic ulcer at the denture-contact point. Excisional biopsy showed hyperplastic stratified squamous epithelium forming fingerlike papillary projections over narrow fibrovascular cores, with parakeratosis, basilar hyperplasia, regular mitotic activity, koilocytes in the spinous layer, and chronic lymphocytic stromal inflammation, with absence of dysplasia or invasion was confirmed. The diagnosis was squamous papilloma with koilocytic change suggestive of HPV cytopathic effect and chronic denture-associated inflammation. These findings supported a diagnosis of squamous papilloma with HPV-suggestive cytopathic effect in the setting of chronic denture-associated irritation.

**Management and outcomes:**

The lesion was completely excised, followed by denture adjustment and reinforcement of oral hygiene measures. Healing was uneventful at one-month review; longer-term follow-up is ongoing.

**Conclusions:**

This case highlights a rare oral squamous papilloma at an atypical mandibular alveolar site, demonstrating overlapping reactive denture-induced and HPV-associated epithelial changes. Histopathological evaluation, particularly koilocyte identification, is essential for diagnosis. Supported by the literature, this case underscores that chronic denture irritation does not preclude an HPV-related etiology, and clinicians should maintain a broad differential for papillary lesions at prosthesis-bearing sites.

## 1 Introduction

Oral squamous papilloma (OSP) is one of the more common benign epithelial lesions of the oral mucosa. It is usually attributed to low-risk HPV, most often genotypes 6 and 11 (1–5). In the landmark series of Abbey and colleagues, OSP accounted for a substantial fraction of all oral soft-tissue biopsies, with the palatal complex, tongue dorsum and lateral borders, and lower lip emerging as the predominant anatomical sites (1). Large contemporary series have reproduced these findings and have additionally emphasized a peak incidence between the third and fifth decades of life, with a slight male predilection (4, 5).

Clinically, OSP usually presents as a solitary, painless, slowly growing, pedunculated or sessile exophytic mass with a characteristic cauliflower-like or fingerlike surface architecture (4, 6). Histologically, the diagnosis rests on the identification of papillary projections of hyperplastic stratified squamous epithelium supported by narrow fibrovascular connective-tissue cores, with variable keratinization, basilar hyperplasia, and, when HPV is implicated, koilocytotic change in the spinous layer (2, 6–8). Koilocytes, which are squamous cells characterized by small hyperchromatic nuclei surrounded by perinuclear halos, remain the most widely accepted light-microscopic surrogate of productive papillomavirus infection, although their sensitivity and specificity for HPV in the oral cavity are imperfect (3, 6, 9).

Denture-related mucosal lesions constitute a separate but occasionally overlapping category of oral pathology. Chronic low-grade mechanical irritation from ill-fitting or inadequately cleaned removable prostheses has long been associated with a spectrum of reactive lesions, including denture stomatitis, inflammatory papillary hyperplasia of the palate, epulis fissuratum, and denture irritation hyperplasia of the alveolar and vestibular mucosa (10–12). These reactive lesions are usually treated as a separate category from HPV papillomas. However, there is evidence that chronic mucosal injury can promote local HPV replication and may help papillomas appear at sites where they are otherwise rare (7, 13).

The anterior mandibular alveolar mucosa is a rare location for OSP; most mandibular exophytic growths beneath the denture border are instead reactive fibrous hyperplasias (12). The present case contributes to the existing literature by documenting a clinicopathologically distinctive presentation in which a histologically typical squamous papilloma with prominent koilocytic change developed at an anatomically uncommon site directly associated with chronic denture irritation (11, 13, 14). This overlap creates a potential diagnostic pitfall, as such lesions may be clinically misinterpreted as purely reactive entities, including epulis fissuratum or denture irritation hyperplasia (2, 11, 15). The identification of koilocytes in this context is therefore of particular diagnostic significance (13, 15).

## 2 Case presentation

### 2.1 Patient information

A 49-year-old male from Syria was referred in April 2026 to our dental clinic for evaluation of an enlarging intra-oral mass of approximately twelve months' duration. The patient reported an absence of systemic illnesses, regular medications, known drug allergies, previous oral surgical procedures, and family history of head-and-neck malignancy. He explicitly denied current or past tobacco use (in any form), alcohol consumption, betel-quid chewing, recreational drug use, or high-risk sexual behavior. He denied a history of HIV/AIDS or other immunosuppressive conditions. He had worn a removable mandibular denture for several years and acknowledged inadequate daily cleaning of its fitting surface. The patient was otherwise in good general health. Informed written consent for publication, including the use of clinical and histological images in anonymized form, was obtained prior to manuscript preparation.

### 2.2 Clinical findings

**Figure 1:**
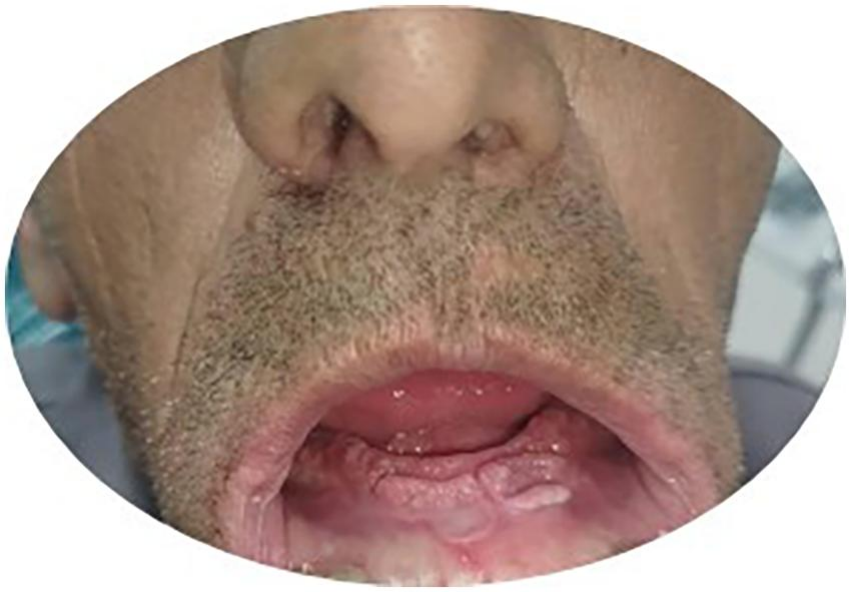
Intra-oral clinical photograph at presentation. Multiple firm papillary mucosal folds of near-normal color are visible on the anterior labial aspect of the mandibular alveolar ridge, directly beneath the flange of the removable denture. A small, adjacent traumatic ulcer (not biopsied) is visible at the denture-contact point.

Extra-oral examination was unremarkable. Absence of facial asymmetry, palpable cervical or submandibular lymphadenopathy, trismus, and motor or sensory deficit in the distribution of the mental nerve was noted. Intra-oral examination revealed a cluster of firm, papillary mucosal folds occupying the anterior labial aspect of the mandibular alveolar ridge mucosa, in the region directly beneath the flange of the removable denture (Figure 1). The lesion was pink to near-normal in mucosal color, measured approximately 25 × 10 mm at its greatest dimensions, was sessile, non-tender to palpation, of near-normal mucosal color, and showed absence of spontaneous bleeding or exudate. A small, superficial traumatic ulcer was identified immediately adjacent to the papillary lesion, corresponding in position to the most rigid part of the overlying prosthesis. The inner fitting surface of the denture demonstrated a visible biofilm deposit and was not cleaned at presentation.

### 2.3 Timeline

The patient first noticed a small painless papillary change beneath the mandibular denture in approximately April 2025. The lesion enlarged slowly and asymptomatically over the following twelve months. He was referred to our department in April 2026 (Day 0), at which time clinical assessment, photographic documentation, and excisional biopsy under local anesthesia were performed in a single visit. The histopathology report was issued on Day +6. Primary healing was uneventful (Days + 7–+ 14), the denture was relined, and oral-hygiene counselling was delivered. Review at Day + 30 showed complete mucosal healing without recurrence. Further reviews are scheduled at 3, 6, and 12 months. A complete CARE-format timeline is provided in Supplementary Table S1.

### 2.4 Diagnostic assessment

#### 2.4.1 Differential diagnosis

The pre-operative working differential diagnosis included: oral squamous papilloma; verruca vulgaris; condyloma acuminatum; focal epithelial hyperplasia (Heck disease); inflammatory fibrous hyperplasia/epulis fissuratum of the alveolar mucosa; verruciform xanthoma; oral verrucous leukoplakia or proliferative verrucous leukoplakia; and given the chronic exposure to a poorly cleaned prosthesis and the duration of the lesion, the more serious possibility of a papillary variant of oral squamous cell carcinoma. An excisional biopsy was therefore considered both diagnostic and therapeutic (2, 3, 5, 6, 15).

#### 2.4.2 Diagnostic procedure

Under aseptic conditions and local infiltration anesthesia, the entire papillary mass was excised down to the submucosa, with a narrow margin of clinically unaffected mucosa. Hemostasis was achieved and the wound was closed with resorbable sutures. The adjacent small ulcer was not sampled, as its clinical appearance was consistent with direct prosthetic trauma and it was expected to resolve following denture correction. The excised specimen was fixed in 10% neutral-buffered formalin, routinely processed, paraffin-embedded, sectioned at 4 *μ*m, and stained with hematoxylin and eosin for light-microscopic assessment. Ancillary molecular HPV typing (polymerase chain reaction or *in situ* hybridization) and immunohistochemistry (p16, Ki-67) were absent from the diagnostic work-up; this limitation is addressed transparently in the Discussion.

#### 2.4.3 Macroscopic description

The specimen was excised in continuity and bisected at the laboratory for processing; two fragments were therefore submitted for histology, together measuring 27 × 12 × 8 mm in their largest dimensions. The external surface was irregular and papillary; on serial sectioning the specimen was uniformly soft, with an absence of grossly identifiable irregular, cystic, or necrotic areas.

#### 2.4.4 Microscopic description

**Figure 2:**
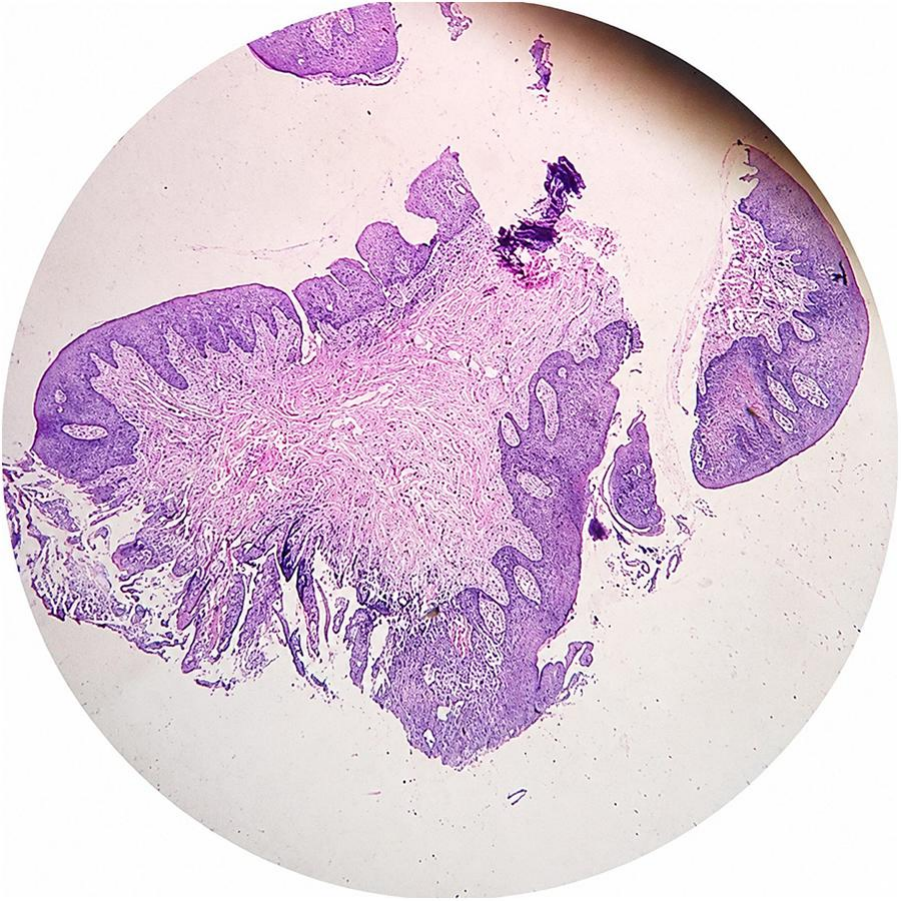
Hematoxylin and eosin, × 4. Scanning-power view of the excised specimen showing the overall papillary architecture: exophytic projections of hyperplastic stratified squamous epithelium supported by narrow, branching fibrovascular connective-tissue cores. Surface parakeratosis is evident.

**Figure 3:**
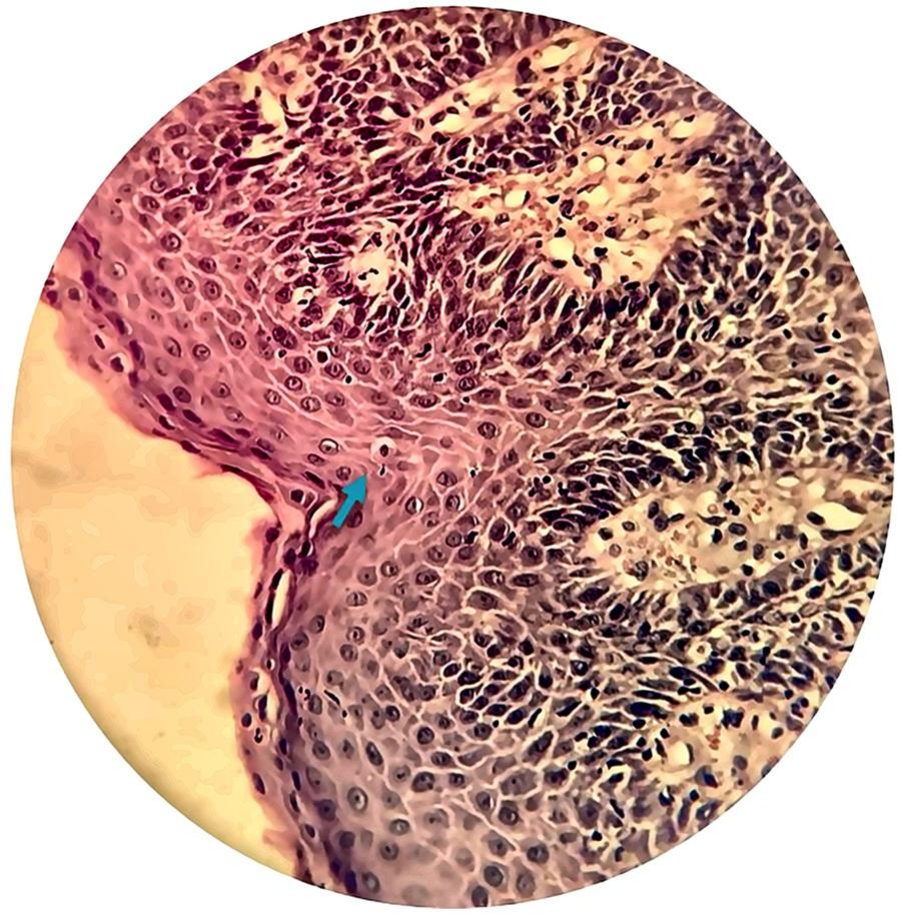
Hematoxylin and eosin, × 40. Well-developed koilocytes are visible high in the spinous layer (teal arrow), characterized by small, hyperchromatic, eccentric nuclei surrounded by clearly defined perinuclear halos, suggestive of human papillomavirus-related cytopathic effect.

Scanning magnification demonstrated multiple slender, fingerlike papillary projections of markedly hyperplastic stratified squamous epithelium supported by narrow, branching, fibrovascular connective-tissue cores containing prominently dilated capillary channels (Figures 2, 3). The surface epithelium showed parakeratosis without significant orthokeratosis. At higher magnification the basal and parabasal zones displayed acanthotic basilar hyperplasia and increased but regular mitotic activity, with an absence of abnormal mitotic figures, dyskeratosis, or full-thickness architectural disarray (Figure 3). The spinous layer contained numerous virus-altered keratinocytes (koilocytes), characterized by small, hyperchromatic, eccentrically placed nuclei surrounded by well-defined perinuclear clear halos, suggestive of an HPV cytopathic effect (Figure 3, arrows). The underlying fibrovascular connective tissue exhibited a moderate, predominantly lymphocytic, chronic inflammatory infiltrate (Figure 3). Absence of cytological atypia, basement-membrane breach, keratin-pearl formation, and evidence of stromal or vascular invasion was noted. Neoplastic transformation was absent.

#### 2.4.5 Pathological diagnosis

Squamous papilloma of the anterior mandibular alveolar mucosa with koilocytic change suggestive of HPV cytopathic effect and chronic denture-associated inflammation.

### 2.5 Therapeutic intervention

Management combined definitive surgical excision with removal of the underlying prosthetic irritant. Complete excisional biopsy served a dual curative and diagnostic purpose, in line with the consensus that surgical excision with a narrow margin is the treatment of choice for OSP and is associated with very low rates of recurrence (1, 4, 5). The patient's existing removable denture was then assessed by the prosthodontic team; the fitting surface was professionally cleaned and the prosthesis was relined to reduce mucosal trauma. Structured oral and prosthetic hygiene instructions were delivered, including nightly removal of the denture, mechanical brushing with a non-abrasive denture brush, immersion in a chemical denture cleanser, and thorough rinsing prior to re-insertion. Smoking and alcohol counselling was not required as the patient was a lifelong non-smoker and non-drinker. An absence of pharmacological adjunct (e.g., topical antifungal therapy or cidofovir) was prescribed, and an absence of laser or cryotherapy was employed; management was deliberately kept conservative and conventional.

### 2.6 Follow-up and outcomes

The patient had an uneventful post-operative course, with an absence of signs of complications during follow-up. At the one-month follow-up review, the alveolar mucosa had re-epithelialized completely, with an absence of residual mass, ulceration, and pain or recurrence at 1-month follow-up. The adjacent denture-related traumatic ulcer had resolved after prosthetic adjustment. The patient reported full acceptance of the new denture hygiene regimen and denied any recurrence of the papillary change. A structured surveillance schedule was agreed upon, comprising clinical re-examination at 3, 6 and 12 months post-excision, with subsequent annual review; the patient was explicitly counselled that mucosal HPV infection may persist subclinically and that any new papillary or white lesion should prompt review (3, 7, 16). No adverse event related to the intervention was recorded.

## 3 Discussion

A review of the literature indicates that oral squamous papilloma (OSP) most frequently arises in the palatal complex, tongue, and lips, with only a small proportion of cases reported in the gingival or alveolar mucosa (2, 14, 15). Large clinicopathological series consistently demonstrate this anatomical predilection, highlighting the rarity of mandibular alveolar involvement (14, 16). Furthermore, while OSP is strongly associated with low-risk human papillomavirus (HPV), particularly genotypes 6 and 11 (2, 13–16), the interaction between HPV-related epithelial changes and chronic mechanical irritation from removable prostheses remains insufficiently characterized (11, 14–17). Only a limited number of reports have described lesions demonstrating overlapping reactive and HPV-associated features, particularly at atypical sites (13, 15).

Four features of this case are worth discussing. The site, the anterior mandibular alveolar mucosa, is rarely reported for OSP. Moreover, the notably large size of the lesion relative to previously reported cases in the literature represents an additional distinguishing feature that, alongside this unusual anatomical site of presentation, further contributes to the novelty of this report. The patient had none of the usual risk factors (he was a lifelong non-smoker and non-drinker). And the lesion combined chronic denture-related irritation with koilocytes that suggested an HPV effect. We discuss each in turn below.

Atypical anatomical location. In the series of Abbey et al. (*n* = 464 oral squamous papillomas), the palatal complex (hard palate, soft palate, and uvula) accounted for 34.3% of cases and was the predominant site, followed in descending order by the dorsum and lateral borders of the tongue and the lower lip; the gingival and alveolar mucosa together contributed only a small minority of cases (1). Bao et al. (*n* = 141 patients from eastern China) reported a similar anatomical predilection, with the palate involved in 41.8% of cases and the tongue in 28.4%, at a mean age of 51.0 years and a male-to-female ratio of 1.82: 1. The anterior mandibular alveolar mucosa is therefore an under-represented site in published series, supporting the clinicopathological interest of the present case (4). Betz, in a comprehensive review of HPV-related oral papillary lesions, confirmed that the soft palate, uvula, tongue, lips, and gingiva account for the great majority of cases (5). A squamous papilloma of the anterior mandibular alveolar mucosa with HPV-suggestive koilocytic change, such as the one reported here, is therefore clinicopathologically uncommon and is liable to be misinterpreted clinically as a purely reactive denture-related fibrous hyperplasia (epulis fissuratum) (12, 18).

Oral squamous papilloma (OSP) is classically described as a small, well-circumscribed exophytic lesion, with the majority of reported cases measuring less than 1 cm in greatest diameter. In the seminal clinicopathological series by Abbey et al., encompassing 464 cases, most lesions were of limited size, consistent with the benign and slow-growing nature of OSP (1). Subsequent reviews have reinforced this characterization, describing OSP as a pedunculated or sessile growth of modest dimensions that rarely exceeds 1 cm (2, 5). Bao et al., in their clinicopathological study of 197 patients, similarly documented that the overwhelming majority of OSPs presented as small lesions, further consolidating limited size as a defining clinical feature of this entity (4). The lesion in the present case measured approximately 25 × 10 mm at its greatest dimensions, placing it well beyond the size range typically reported in the literature. When lesions deviate substantially from this expected size, the differential diagnosis becomes considerably more demanding, as larger exophytic papillary growths of the oral mucosa raise concern for entities such as papillary squamous cell carcinoma, verrucous carcinoma, or condyloma acuminatum, all of which may share overlapping morphological features with OSP (5, 12, 13). This atypically large size, combined with the unusual anatomical location described above, collectively reinforces the clinical novelty of this case and highlights the critical importance of histopathological examination when encountering unexpectedly large papillary oral lesions that may otherwise appear benign on clinical grounds alone (12, 13).

Absence of tobacco and alcohol exposure. The patient denied tobacco and alcohol use, reducing the likelihood of conventional carcinogen-associated oral epithelial change; however, the absence of these exposures doesn't exclude HPV-related or reactive papillary lesions (7, 15). In this case, the clinicopathological findings supported a lesion with overlapping chronic denture-associated irritation and possible HPV-related cytopathic change rather than a primary dysplastic or invasive process. Proliferative verrucous leukoplakia and papillary squamous cell carcinoma were therefore considered in the differential diagnosis, but histopathological exclusion of these entities remained essential (4, 6, 9).

Co-existence of reactive and viral features. The histopathological picture in this case is instructive. On the one hand, the fibrovascular stroma, dilated capillaries, chronic lymphocytic infiltrate, and adjacent traumatic ulcer all conform to the well-described morphology of denture-related reactive hyperplasias such as epulis fissuratum and inflammatory papillary hyperplasia, which are strongly associated with ill-fitting or poorly maintained prostheses and tend to regress partially after removal of the irritant (10–12). On the other hand, the fingerlike papillary projections, parakeratosis, basilar hyperplasia, and most importantly the unequivocal koilocytes in the spinous layer are cardinal features of an HPV-suggestive squamous papilloma (2, 3, 6). Koilocytosis is widely regarded as the most specific light-microscopic marker of productive low- and, less often, high-risk HPV infection, even though its sensitivity may be modest and genotype cannot be inferred from morphology alone (3, 6). It is biologically plausible that chronic denture-related trauma and associated biofilm accumulation acted synergistically to disrupt mucosal integrity, thereby permitting a latent HPV infection to become morphologically evident in the compromised epithelium (15).

Differential diagnosis and diagnostic challenges. A useful clinicopathological comparison of the key differential diagnoses is provided in Table 1. Clinically, denture irritation hyperplasia typically presents as one or more smooth fibrous folds accommodating the denture flange, with minimal papillary architecture and an absence of koilocytes on histology (12). Inflammatory papillary hyperplasia almost always affects the hard palate of a long-standing denture wearer and, while papillary, lacks viral cytopathic effect (10, 11). Verrucous leukoplakia and condyloma acuminatum can mimic OSP but usually exhibit more marked hyperkeratosis with prominent granular-layer change, coarse keratohyalin granules and cup-shaped endophytic growth (verruca), or broader-based exophytic growth with acanthosis and abundant koilocytes (condyloma) (5, 6, 8, 15). Importantly, condyloma acuminatum was considered unlikely in the present case because the lesion was solitary rather than multifocal, arose at a denture-bearing site with a documented history of chronic mechanical irritation, and the patient explicitly denied orogenital contact or high-risk sexual behavior. Histologically, condyloma typically demonstrates broader, rounded, bulbous papillary projections with less prominent fibrovascular cores and more diffuse acanthosis, whereas the present lesion showed slender, fingerlike projections supported by narrow, branching fibrovascular cores with parakeratosis—features more consistent with OSP (5, 6, 8, 15). Verruciform xanthoma is excluded by the absence of xanthomatous (foamy) cells in the papillary stroma. The most clinically important mimicker is papillary squamous cell carcinoma, which may likewise present as an exophytic cauliflower-like lesion at a denture-bearing site but shows full-thickness dysplasia, significant cytological atypia, and frankly infiltrative architecture (4, 12). Careful orientation of the specimen and assessment of the base of the papillary projections are essential to avoid under-diagnosis.

**Table 1:** Principal clinicopathological differential diagnoses for a denture-associated papillary lesion of the alveolar mucosa.

Entity	Typical site	Key histological clues	Relation to the present case
Oral squamous papilloma (HPV 6/11)	Palate, tongue, lips, uvula; alveolar mucosa rare	Papillary projections; fibrovascular cores; parakeratosis; koilocytes in spinous layer; no dysplasia	Final diagnosis in this case
Verruca vulgaris (HPV 2/4, 1, 57)	Labial mucosa, anterior tongue	Pronounced hyperkeratosis; cup-shaped endophytic growth; coarse keratohyalin granules; koilocytes	Excluded, lesion was predominantly exophytic with parakeratosis and without cup-shaped base
Condyloma acuminatum (HPV 6/11)	Soft palate, ventral tongue, labial mucosa	Broad-based, blunted papillae; marked acanthosis; abundant koilocytes	Excluded, papillae in this case were slender and fingerlike rather than broad and blunted
Focal epithelial hyperplasia (Heck disease, HPV 13/32)	Labial and buccal mucosa, often multifocal	Acanthosis; mitosoid bodies; limited keratinization; predominantly plaque-like, not papillary	Excluded, solitary, unequivocally papillary lesion; no mitosoid figures
Inflammatory papillary hyperplasia (Typically not associated with HPV)	Almost exclusively hard palate of denture wearers	Multiple small papillary projections; pseudo-epitheliomatous hyperplasia; chronic inflammation; no koilocytes	Excluded clinically by mandibular alveolar site; excluded histologically by koilocytosis
Epulis fissuratum/denture irritation hyperplasia (Typically not associated with HPV)	Maxillary/mandibular vestibule along denture flange	Fibrous hyperplasia with elongated rete ridges; pseudo-epitheliomatous. hyperplasia may occur; no koilocytes. Usually smooth or lobulated, not finger-like	Partially overlapping clinically (denture association) but lacks viral cytopathic effect
Verruciform xanthoma (Not associated with HPV)	Gingiva, alveolar ridge	Papillary surface; parakeratosis; foamy xanthoma cells within papillary stroma	Excluded, no xanthoma cells identified
Papillary squamous cell carcinoma (HPV-16, HPV-18 high-risk HPV types)	Gingiva, alveolar ridge, tongue in older patients	Full-thickness dysplasia; atypical mitoses; infiltrative base; desmoplastic stroma	Excluded, there is no dysplasia, atypia, or invasion

Final discrimination from papillary squamous cell carcinoma requires careful assessment of the lesion base to identify full-thickness dysplasia, atypical mitoses, and infiltrative architecture.

Management of OSP is generally straightforward. Complete surgical excision is both diagnostic and curative in most cases, with recurrence being uncommon (1, 4, 5, 14). In this patient, treatment also involved addressing the underlying denture-related issue by improving fit and reinforcing hygiene practices. This is an important aspect of care, as ongoing mechanical irritation and poor denture hygiene are well-recognized contributors to persistent mucosal changes (10–12). The favorable short-term outcome in this case, with complete healing and no recurrence at one month, is consistent with what would be expected following appropriate management.

Even so, follow-up remains important. Although recurrence rates are low, they are not negligible, and lesions arising at unusual sites or in the presence of ongoing local factors may warrant closer observation (13, 16). Patient education is also essential, particularly in relation to denture hygiene and the need to seek evaluation if new lesions develop.

### 3.1 Strengths and limitations

The strength of this report is that the clinical, prosthetic and histopathological assessment was carried out together rather than in isolation, and the case was written up against the CARE checklist (19, 20). Nevertheless, several limitations must be acknowledged. First, the intra-oral photograph (Figure 1) was obtained from a relatively external perspective with the patient's mouth only partially open, limiting full appreciation of the lesion's extent, surface texture, and precise relationship to the denture flange; an intra-oral close-up with the denture removed, ideally using a mouth mirror or retractor to expose the alveolar mucosa fully, was not obtained at the time of presentation and is acknowledged as a limitation of the clinical documentation. Second, molecular confirmation of HPV genotype was absent; polymerase chain reaction or *in situ* hybridization would have enabled formal discrimination between low- and high-risk HPV subtypes and is recommended for future similar cases, in keeping with the broader literature (2, 3, 5, 13). Third, additional immunohistochemistry (notably p16 as a surrogate for transcriptionally active high-risk HPV, and Ki-67 to exclude high proliferative index) was absent from the diagnostic work-up; although the morphological criteria for a benign koilocytic papilloma were strongly supportive, such studies would have strengthened pathological confidence. Fourth, follow-up is still early and long-term recurrence data are not yet available; nonetheless, a structured surveillance plan has been put in place. Despite these limitations, the case remains clinically and educationally valuable, because the simultaneous presence of denture-related and HPV-related features at an atypical anatomical site is rarely documented with both clinical and histological images in the peer-reviewed literature.

From a clinical perspective, this case underscores the importance of maintaining a broad differential diagnosis when evaluating papillary lesions in denture-bearing areas (2, 11). While reactive lesions remain the most common entities in such locations, the possibility of HPV-associated papilloma should not be overlooked, particularly when lesions exhibit exophytic or papillary architecture (13, 15). Failure to recognize this distinction may lead to underdiagnosis or inappropriate management (13, 15). Therefore, histopathological examination remains essential for definitive diagnosis (13–15).

**Figure 4:**
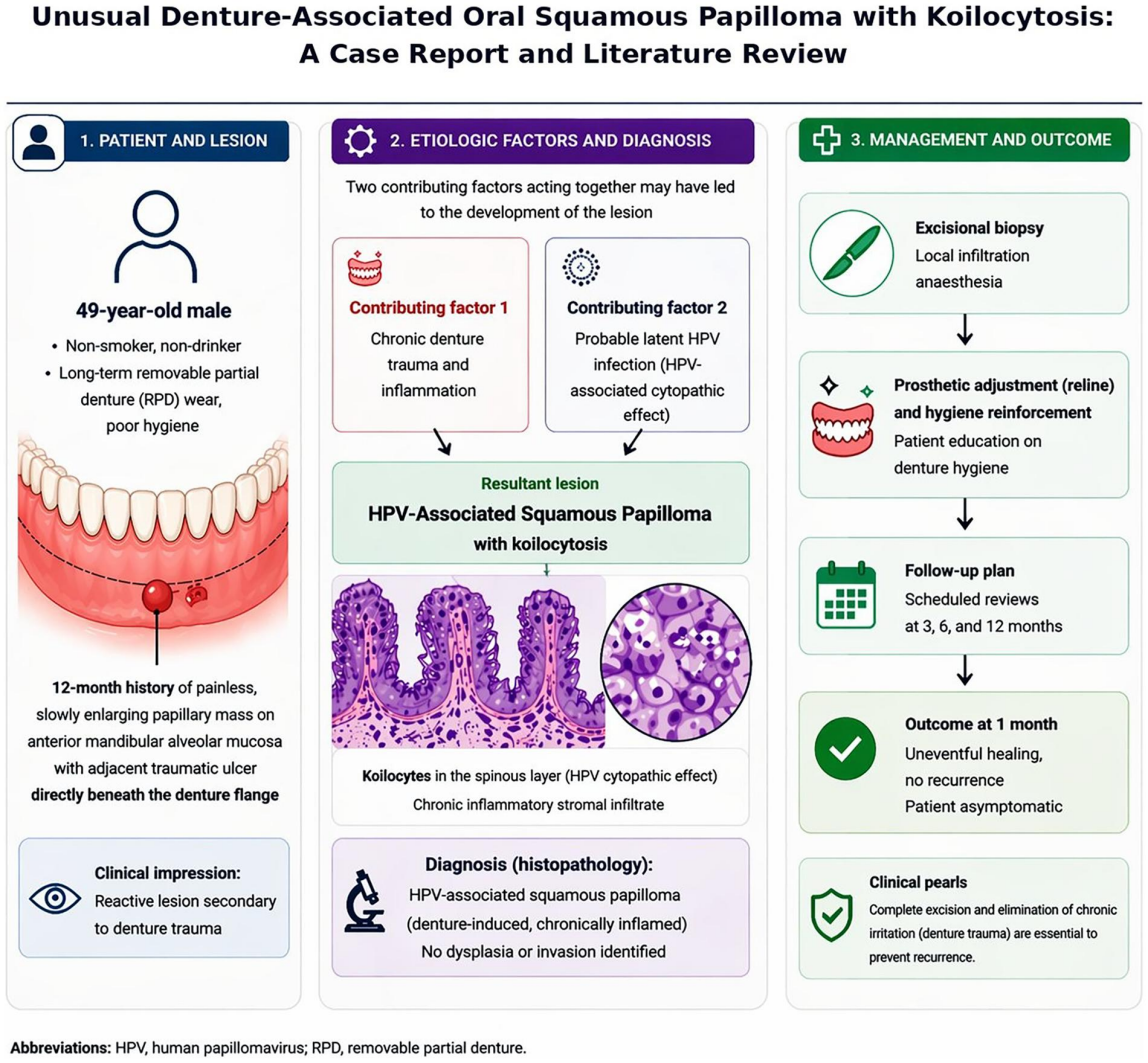
Graphical abstract. Visual summary of the case illustrating the clinical presentation of a papillary lesion at the anterior mandibular alveolar mucosa beneath a removable denture flange, key histopathological features (papillary projections, koilocytes with perinuclear halos), and the management pathway. The arrow indicates the koilocytic change in the spinous layer.

Overall, the available literature suggests that although OSP is a common benign lesion, its occurrence at atypical anatomical sites in association with chronic mechanical irritation is uncommon and likely underreported (2, 13–15). The present case adds to the limited body of evidence supporting a potential interaction between local irritative factors and HPV-related epithelial proliferation, highlighting the need for further clinicopathological and molecular studies (2, 15, 16). A visual summary of the key clinical, histopathological, and management features is presented in (Figure 4).

## 4 Conclusions

Oral squamous papilloma with HPV-suggestive koilocytic change should be considered in the differential diagnosis of any chronic papillary mucosal lesion in denture wearers, even in the absence of classical tobacco and alcohol risk factors and even when the anatomical site is atypical. When reactive denture changes and HPV cytopathic effect appear in the same lesion, the diagnosis can easily be missed. The koilocytes in the spinous layer are what give it away, so they need to be looked for specifically. Definitive management combines complete surgical excision with correction of the underlying prosthetic irritant and robust patient-centered denture-hygiene instruction. HPV genotyping by PCR or *in situ* hybridization, along with p16 immunohistochemistry, is not available everywhere, but it is worth ordering when the picture is ambiguous or when dysplasia is suspected. Long-term follow-up should still be arranged in either case.

## Data Availability

The raw data supporting the conclusions of this article will be made available by the authors, without undue reservation.
