# Supplementary material for: Unusual denture-associated oral squamous papilloma with koilocytosis: a case report and literature review
Source: Front Dent Med. 2026 Jun 15;7:1871334. doi: 10.3389/fdmed.2026.1871334 (PMC13310973; doi:10.3389/fdmed.2026.1871334)
Supplement: Supplementary file 1 [file Datasheet1.pdf]

Supplementary Material

A concise chronological summary of the diagnostic and therapeutic events is presented in Table S1.

Date (approximate)	Clinical event / intervention
**~April 2025**	Patient first noticed a small, painless papillary mucosal change beneath the mandibular denture; no dental consultation at that time.
**January - March 2026**	Slow enlargement of the lesion over several months; patient remained asymptomatic; denture wear continued without adequate hygiene.
**April 2026 (Day 0)**	Referral to the Department of Oral Medicine, AIU; intra-oral examination and full clinical documentation; working differential diagnosis formulated.
**Day 0 (same visit)**	Excisional surgical biopsy of the papillary lesion performed under local infiltration anesthesia; specimen submitted for histopathological assessment; denture-hygiene counselling initiated.
**Day +6**	Histopathological report issued: squamous papilloma with koilocytic change suggestive of HPV cytopathic effect and chronic denture-associated inflammation.
**Day +7 to Day +14**	Uneventful primary healing; denture rebasing/relining arranged; the patient was educated on proper denture hygiene, including nightly removal, mechanical brushing, and chemical immersion.
**Day +30 (first follow-up)**	No clinical evidence of recurrence; mucosa fully healed; patient asymptomatic.
**Planned: 3-, 6-, 12-month follow-ups**	Scheduled clinical re-examination for early detection of any recurrence or new lesion; long-term denture-hygiene adherence monitoring.

Table S1. Timeline of clinical events, diagnostic assessment, intervention, and follow-up.

Table S2: CARE Checklist Mapping

The table below maps each CARE checklist item to the corresponding section of this manuscript, in accordance with the 2013 consensus statement (15) and the 2017 Explanation and Elaboration document (16).

CARE item	Topic	Where addressed
**1**	Title - diagnosis and CARE adherence indicated	Title page
**2**	Key words	Abstract (end)
**3**	Abstract (structured) - background, case, conclusions	Abstract
**4**	Introduction - background, literature context	Section 1
**5a**	Patient information - demographics, history, relevant comorbidities	Section 2.1
**5b**	Main complaint and presenting concern	Sections 2.1-2.2
**6**	Clinical findings - relevant physical and clinical examination findings	Section 2.2
**7**	Timeline - historical and current information in chronological order	Section 2.3 and Table 1
**8a**	Diagnostic assessment - diagnostic methods, challenges, reasoning	Section 2.4
**8b**	Diagnostic reasoning including differential diagnosis	Section 2.4 and Table 2
**9a**	Therapeutic intervention - types and details	Section 2.5
**9b**	Changes in intervention with rationale	Section 2.5 (prosthetic correction and hygiene regimen)

**10**	Follow-up and outcomes - clinician- and patient-assessed outcomes, adverse events	Section 2.6
**11**	Discussion - strengths, limitations, comparison with literature, take-away lessons	Section 3
**12**	Patient perspective	Section 5
**13**	Informed consent	Section 6 (Declarations)
